# Quality of reporting web-based and non-web-based survey studies: What authors, reviewers and consumers should consider

**DOI:** 10.1371/journal.pone.0194239

**Published:** 2018-06-18

**Authors:** Tarek Turk, Mohamed Tamer Elhady, Sherwet Rashed, Mariam Abdelkhalek, Somia Ahmed Nasef, Ashraf Mohamed Khallaf, Abdelrahman Tarek Mohammed, Andrew Wassef Attia, Purushottam Adhikari, Mohamed Alsabbahi Amin, Kenji Hirayama, Nguyen Tien Huy

**Affiliations:** 1 Faculty of Medicine, Damascus University, Damascus, Syria; 2 Zagazig University Hospital, Department of Pediatrics, Sharkia, Egypt; 3 Harvard T.H Chan School of Public Health, Boston, United States of America; 4 Medical Microbiology and Immunology Department, Faculty of Medicine, Tanta University, Tanta, Egypt; 5 Maternity and Children Hospital, Makkah, Saudi Arabia; 6 Faculty of Medicine, Cairo University, Cairo, Egypt; 7 Faculty of Medicine, Alazhar University, Cairo, Egypt; 8 Ain Shams University Hospitals, Cairo, Egypt; 9 Gandaki Medical College, Pokhara, Nepal; 10 Zagazig University Hospital, Department of Plastic Surgery, Sharkia, Egypt; 11 Department of Immunogenetics, Institute of Tropical Medicine (NEKKEN), Graduate School of Biomedical Sciences, Nagasaki University, Sakamoto, Nagasaki, Japan; 12 Evidence Based Medicine Research Group & Faculty of Applied Sciences, Ton Duc Thang University, Ho Chi Minh City, Vietnam; 13 Department of Clinical Product Development, Institute of Tropical Medicine (NEKKEN), Nagasaki University, Sakamoto, Nagasaki, Japan; Cardiff University, UNITED KINGDOM

## Abstract

**Background:**

Several influential aspects of survey research have been under-investigated and there is a lack of guidance on reporting survey studies, especially web-based projects. In this review, we aim to investigate the reporting practices and quality of both web- and non-web-based survey studies to enhance the quality of reporting medical evidence that is derived from survey studies and to maximize the efficiency of its consumption.

**Methods:**

Reporting practices and quality of 100 random web- and 100 random non-web-based articles published from 2004 to 2016 were assessed using the SUrvey Reporting GuidelinE (SURGE). The CHERRIES guideline was also used to assess the reporting quality of Web-based studies.

**Results:**

Our results revealed a potential gap in the reporting of many necessary checklist items in both web-based and non-web-based survey studies including development, description and testing of the questionnaire, the advertisement and administration of the questionnaire, sample representativeness and response rates, incentives, informed consent, and methods of statistical analysis.

**Conclusion:**

Our findings confirm the presence of major discrepancies in reporting results of survey-based studies. This can be attributed to the lack of availability of updated universal checklists for quality of reporting standards. We have summarized our findings in a table that may serve as a roadmap for future guidelines and checklists, which will hopefully include all types and all aspects of survey research.

## Introduction

Surveys are powerful research tools that convey valuable information on disease trends, risk factors, treatment outcomes, quality of life, and cost-effectiveness of care.[1, 2] Moreover, from a methodological standpoint, surveys facilitate having a larger sample size and therefore a greater statistical power, increase the ability of gathering large amounts of information, increase the accessibility to targeted population by using several online and offline modes of administrations, and promote the usage of validated tools of measurement.[3]

The high influx of survey data in our contemporary and fast-paced scientific world highlights the need to critically assess the usefulness and validity of research findings. This emphasizes the importance of survey reporting guidelines. Rigorous reporting prevents misinterpretations and improper applications that might bring harm to patients. It can also help editors and reviewers maintain a focused high-quality review process.[4, 5] Hence, there is an increasing need for journals endorsing those guidelines and referring authors to using core specialized reporting checklists that best serve their study design.[6]

It is well-established that following reporting guidelines improves the quality of reporting of research studies.[7] It provides a framework upon which evidence can be transparently consumed and reproduced.[7] However, several reports have demonstrated that key quality criteria of clinical and survey studies are under-reported.[8–11] In addition, there is no global consensus on the optimal reporting of survey research, and only few medical journals provide guidance to authors regarding the reporting of questionnaire-based projects. Furthermore, the constant development and change of scientific knowledge and research methodology imposes a vital need to continually assess and update current guidelines.[8, 12] For instance, collecting data through web-based surveys is an increasingly popular widely-used research methodology, which still lacks a validated reporting guideline.[13]

Few previous reviews and reports highlighted the importance of reporting some key points in non-web-based survey studies.[14] However, many influential items still need further consideration. We therefore aim through this study to investigate the reporting practices and quality of both web- and non-web-based survey studies, to report any potential shortfalls in current practices, and to reach a crucial milestone in developing a comprehensive guideline to enhance the quality of reporting medical evidence that is derived from survey studies and to maximize the efficiency of its consumption.

## Methods

### Search methods and reference management

**Fig 1:**
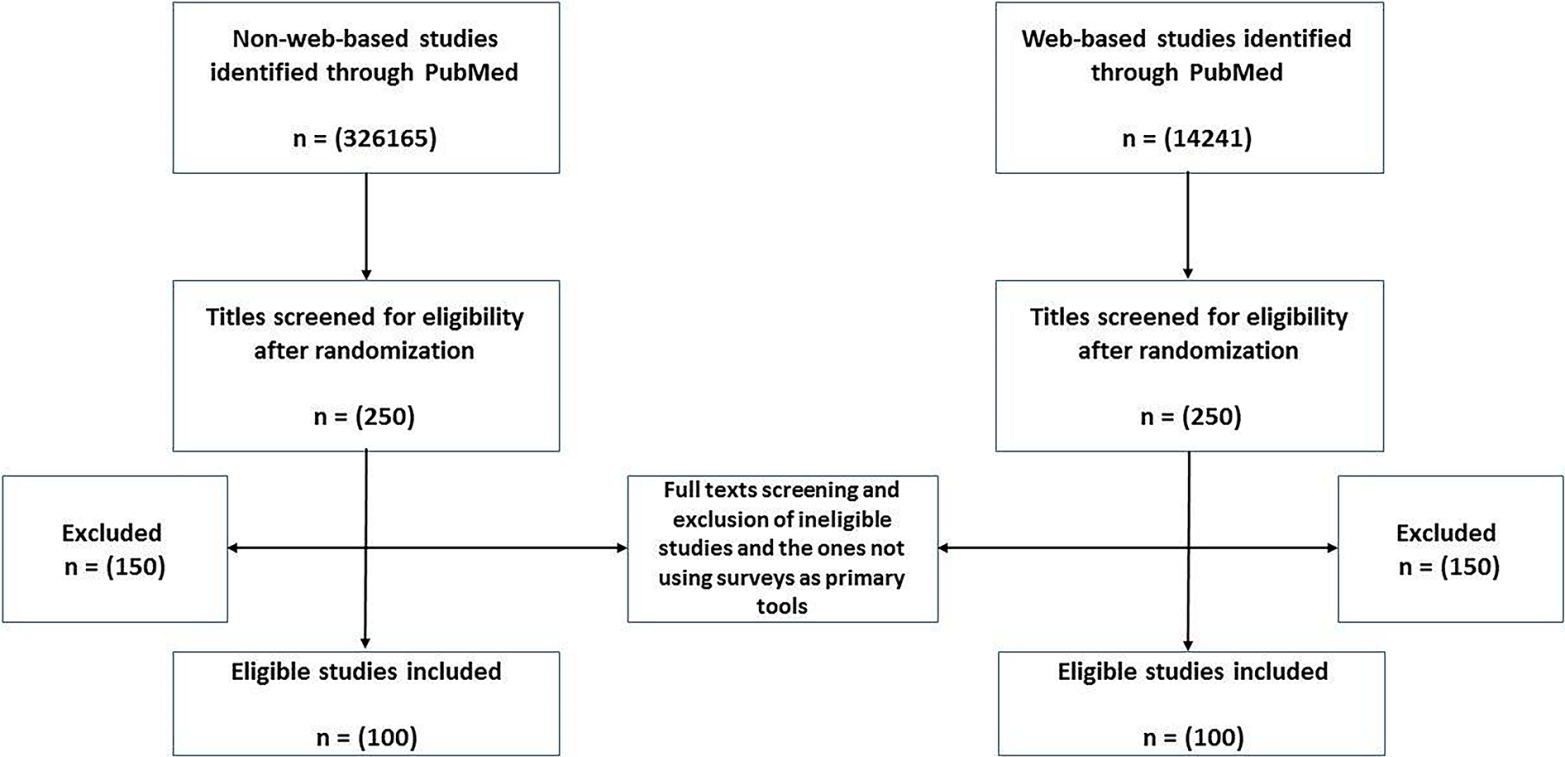
Flow diagram of records and reports.

We have conducted two separate PubMed searches to retrieve web-based and non-web-based survey studies, using the search terms ("web-based" OR "Online") AND ("Survey" OR "Questionnaire") and ("Surveys” OR “Questionnaires"), respectively. We restricted our search to the period between 1\1\2004 and 31\12\2016. Upon retrieving titles and abstracts, we created separate libraries for web- and non-web-based studies (i.e. an excel sheet that contains all titles of all web-based studies, and another one for non-web-based studies). In order to obtain a random sample, we generated random ID numbers for all retrieved references in both libraries using Microsoft Excel. We then sorted the references according to their ID numbers from smallest to largest. Afterwards, we screened the titles to get to the first 250 potentially included papers in each library. Lastly, we screened full texts to get to a 100 finally included studies for each section. The whole procedure is demonstrated in Fig 1.

### Eligibility criteria and study selection

We included all original survey-based studies that were published between 1\1\2004 and 31\21\2016 to assess the reporting quality in these years, and excluded all other types of publications, such as commentaries, letters, reviews, etc., and study designs, such as randomized clinical trials (RCTs), cohort studies and case-control studies, where surveys were only used for demographic data.

Exclusion of these studies followed a consistent and accurate approach to only include original articles that rely mainly on questionnaires to generate their evidence (i.e. we excluded studies that used questionnaire for demographics and only included the ones that are purely survey-based). To select eligible studies, three co-authors independently screened the 250 articles of each pool. Screening at this phase was to determine a study’s eligibility for inclusion into our analysis. All differences in screening were resolved by consensus.

### Tool for reporting quality of non-web-based survey studies

For the non-web-based section of the study, we relied on the SUrvey Reporting GuidelinE (SURGE)[14] to construct a purpose-designed extraction sheet. Top journals like The Lancet, JAMA and PLOS Medicine recommend using the EQUATOR Network’s website (www.equator-network.org) to find the most suitable reporting guideline according to the study design. The EQUATOR Network recommends the STROBE (STrengthening the Reporting of Observationally studies in Epidemiology) Statement (www.strobe-statement.org) as the best reporting guideline available currently. However, the STROBE Statement does not include methods’ and results’ reporting characteristics that are unique to surveys (8). It mainly deals with cross sectional studies that include surveys as a complementary part of the study, and studies that use surveys in the field of epidemiology. We, therefore, searched for a tool that uses the STROBE statement as the core for its development and in the same time focuses on non-web based surveys which was the reason we used SURGE as the main reporting guideline in this study.

We modified a single code in the SURGE guideline to include all possibilities of extractions and insure a more accurate extraction. The modification involves modifying modes of administration to have five codes (In person, Telephone, Mail, Mixed and Not mentioned) instead of the four codes originally used in SURGE (In person, Mail, Mixed and Not explicitly stated) as some of our included surveys used telephone as the mode of administrating the survey and we could not include this under any of the four codes of SURGE guideline. All outcomes are summarized in Table 1.

**Table 1:** Results of assessment of 100 non-web-based studies using Bennett et al.'s SURGE guidelines.

Checklist item	Explanation/coding	Number (%) in our study
**Title and Abstract**		
Design of study stated	Both title and abstract	14
	Either title or abstract	63
	Not stated	23
**Introduction**		
Background provided	Yes	100
	No	0
Purpose/aim of paper explicitly stated	Yes	99
	No	1
**Methods**
**Research Tool**		
Description of the questionnaire	Questionnaire provided	21
	Core questions provided	28
	One complete question provided	13
	Questions not provided	38
Existing tool, psychometric properties presented	Yes	48
	No	27
	Not applicable	25
Existing tool, references to original work provided	Yes	55
	No	20
	Not applicable	25
New tool, procedures to develop and pre-test provided	Yes	30
	No	40
	Not applicable	30
New tool, reliability and validity reported	Both	3
	Reliability only	3
	Validity only	15
	Neither	49
	Not applicable	30
Description of the scoring procedures provided	Yes	56
	No	38
	Not applicable	6
**Sample Selection**		
Description of survey population and sample frame	Both	82
	Survey population	7
	Sample frame	3
	Neither	8
Description of representativeness of the sample	Yes	43
	No	57
Sample size calculation or rationale/justification presented	Yes	37
	No	63
**Survey Administration**		
Mode of administration	In person	43
	Telephone	6
	Mail	17
	Mixed	15
	Not mentioned	19
Information on the type and number of contacts provided	Type and number	18
	Type only	33
	No information	49
Information on financial incentives provided	Yes	21
	No	79
Description of who approached potential participants	Yes	32
	No	68
**Analysis**		
Method of data analysis described	Adequate	79
	Inadequate	15
	No description	6
Method for analysis of nonresponse error provided	Yes	13
	No	87
Method for calculating response rate provided	Yes	32
	No	68
Definitions for complete versus partial completions provided	Yes	11
	No	89
Methods for handling item missing data provided	Yes	21
	No	79
**Results**		
Response rate reported	Yes, defined	32
	Yes, not defined	8
	Partial information	20
	No information	40
All respondents accounted for	Yes	51
	No	49
Information on how non-respondents differ from respondents provided	Yes	8
	Issue addressed	11
	No information	81
Results clearly presented	Yes–complete	87
	Yes–partial	13
	No	0
Results address objectives	Yes	100
	No	0
**Discussion**		
Results summarized referencing study objectives	Yes	100
	No	0
Strengths of the study stated	Yes	46
	No	54
Limitations of the study stated	Yes	75
	No	25
Generalizability of results discussed	Yes	35
	No	65
**Ethical Quality Indicators**		
Study funding reported	Yes	58
	No	42
Research Ethics Board (REB) review reported	Yes	67
	Reported REB exempt	2
	No	31
Subject consent procedures reported	Yes	46
	Reported waiver of informed consent	3
	No	51

### Tool for reporting quality of web-based survey studies

For web-based studies, we used the updated CHERRIES guidelines [15] to construct another purpose-designed extraction sheet. As we previously mentioned, we depended on the EQUATOR Network’s website (www.equator-network.org) to find the most suitable reporting guideline. Among the suggested guidelines we found CHERRIES to be the most suitable guideline considering that we are searching for a tool mainly designed for web-based surveys. The scoring system and outcomes are summarized in Table 2.

**Table 2:** Frequency of reporting in our 100 included web-based survey studies using Eysenbach et al’s CHERRIES guidelines.

Checklist item	Explanation	Number (%)
**Design**		
Describe survey design	Describe target population, sample frame and the sample is a convenience sample	74
	Describe only two of those three points	21
	Describe population only	4
	Describe sample frame only	1
	Describe sample as convenience only	0
	Describe none of the above	0
**IRB (Institutional Review Board) approval and informed consent process**		
IRB approval	Mention that the study has been approved by an IRB	76
	Not mentioned	24
Informed consent	Describe the informed consent process in details (telling the participants how long will the survey take, which data will be stored and where and for how long, who the investigators are, and what the purpose of the study is)	4
	Describe the informed consent process with some of the above details	5
	Just mentioning taking the informed consent	38
	Not mentioning taking the informed consent	53
Data protection	If authors collected or stored any personal information, they gave the mechanisms used to protect unauthorized access.	24
	Mechanisms used are not given	76
**Development and pre-testing**		
Development and testing	State how the survey was developed with testing the usability and technical functionality of the electronic questionnaire.	37
	State how the survey was developed without testing the usability and technical functionality of the electronic questionnaire.	26
	Not mentioning the development process	37
**Recruitment process and description of the sample having access to the questionnaire**		
Open survey versus closed survey	An “open survey” which is a survey open for each visitor of a site.	19
	A closed survey that is only open to a sample that the investigator knows (password-protected survey).	24
	Not clear or not explicitly stating the type of survey	57
Contact mode	The initial contact with the potential participants was through the Internet or e-mail.	73
	The initial contact with the potential participants was through mail while allowing web based data entry	2
	Contact mode was not clear	25
Advertising the survey	Through online mailing lists	49
	Through offline media (newspapers)	7
	Through social media	2
	Through banner ads	1
	Mixed	7
	Not mentioned	34
Wording of the advertisement	Given	1
	Not given	99
**Survey administration**		
Web/E-mail	Survey posted both website and e-mail	1
	Survey posted on website	65
	Survey sent through e-mail	20
	Not clear	14
Data entry in e-mail sent surveys	Manually	3
	Automatic	3
	Not clear	94
Context	Describe the Web site (for mailing list/newsgroup) in which the survey was posted. What is the Web site about, who is visiting it, what are visitors normally looking for? Discuss to what degree the content of the Web site could pre-select the sample or influence the results.	3
	Described in partial details	21
	No information about the website was given or just mentioning its name.	76
Mandatory/ Voluntary	It was a mandatory survey to be filled in by every visitor who wanted to enter the Web site.	1
	It was voluntary	19
	Not clear or not mentioned	80
Incentives	Monetary incentives or prizes were offered.	11
	Non-monetary incentives such as an offer to provide the survey results were offered	11
	Not mentioned or they mentioned not giving any incentives	78
Time/Date	Authors gave the timeframe in which data were collected	68
	Not given	32
Randomization of items of questionnaires	To prevent biases, items can be randomized or alternated	1
	Not randomized or not mentioned	99
Adaptive questioning	Use adaptive questioning to reduce number and complexity of the questions. (Displaying certain items based on responses to other items)	10
	Not used or not mentioned	90
Number of Items	The number of questionnaire items per page was given	3
	Not given or not mentioned	97
Number of screens (pages)	The number of screens (pages) in the questionnaire was given.	3
	Not given or not mentioned	97
Completeness check	Consistency or completeness checks was done before the questionnaire is submitted	4
	Consistency or completeness checks was done after the questionnaire is submitted.	4
	Not mentioned	92
A non-response option	A non-response option such as “not applicable” or “rather not say” was given and enforced.	4
	Not given	96
Review step	Respondents were able to review and change their answers (e.g. through a Back button or a Review step which displays a summary of the responses and asks the respondents if they are correct).	1
	Not provided	99
**Response rates**		
Unique site visitor	The number of unique site visitors was given.	3
	Not given	97
View rate	Requires counting unique visitors to the first page of the survey, divided by the number of unique site visitors (not page views!).	0
	Not given.	100
Participation rate (Recruitment rate)	Count the unique number of people who filled in the first survey page (or agreed to participate, for example by checking a checkbox), divided by visitors who visit the first page of the survey (or the informed consents page, if present).	37
	Not given	63
Completion rate	The number of people submitting the last questionnaire page divided by the number of people who agreed to participate (or submitted the first survey page).	38
	Not given	62
**Preventing multiple entries from the same individual**		
Cookies used	Yes	2
	No/not mentioned	98
How cookies work	preventing users from accessing the survey twice	1
	duplicates got eliminated before analysis and 1st entry got used	0
	duplicates got eliminated before analysis and the last entry got used	0
	Not mentioned	99
IP check	IP check used and the period of time for which no two entries from the same IP address were allowed	0
	IP check used without giving the period of time for which no two entries from the same IP address were allowed	2
	not used or mentioned	98
How IP check was used	preventing users from accessing the survey twice	1
	duplicates got eliminated before analysis and 1st entry got used	0
	duplicates got eliminated before analysis and the last entry got used	0
	Not mentioned	99
Log file analysis	Other techniques to analyze the log file for identification of multiple entries were used.	1
	None used	99
Registration	Describe methods of closing the survey (For example, was the survey never displayed a second time once the user had filled it in, or was the username stored together with the survey results and later eliminated)	6
	Not described	94
**Analysis**		
Handling of incomplete questionnaires	Only completed questionnaires analyzed	19
	Both complete and partial questionnaires analyzed	4
	Not mentioned	77
Questionnaires submitted with an atypical timestamp	The timeframe that was used as a cut-off point was given and described why	0
	The time frame was given but without the reason	3
	Not mentioned	97
Statistical correction	Methods to adjust for the non-representative sample (such as weighting of items or propensity scores) was given and described	6
	Methods to adjust for the non-representative sample was given but not described	0
	not given or mentioned	94

While the original CHERRIES guideline has eight categories with 30 items, our checklist has the same eight categories with an extended 35 checklist items. We added those extra five checklist items from the explanations given by Eysenbach et al. in his article. These five items include the wording of the advertisement, the data entry in e-mail sent surveys, giving a non-response option, telling how cookies work and telling how IP check was used. We added those items to help us better assess the main eight categories, and to achieve a thorough data extraction process.

### Data extraction

Three authors independently extracted data from all included studies, and we divided the extraction process into several cycles. In each cycle, authors extracted 5–10 studies. Each author provided justifications for every criterion assessed and extracted, and at the end of each cycle, resolved all disagreements through discussion. All unresolved or unclear information was discussed with the senior author (NTH) and final decisions were documented. A separate author (TT) then double-checked our data for any entry-errors after each cycle as well as at the end of our analysis.

### Statistical analysis

We reported scores for all items as frequencies and percentages using Microsoft Excel.

## Results

### Quality of reporting in non-web-based surveys using Bennett et al.’s SURGE guidelines

We included 100 non-web-based survey studies using the methodology described in the flow chart in Fig 1. Using Bennett et al.’s SURGE guidelines, we assessed the reporting quality of those 100 survey studies and reported all results in Table 1.

Our results reveal that a lot of the checklist's items are under-reported especially in the analysis section. Aside from describing the methods of data analysis which was adequate in 79% of the papers, 87% of the studies did not provide methods for analyzing the non-response error, 68% did not tell how response rate was calculated, 89% did not provide definitions for complete and partially complete questionnaires and 79% did not tell how they handled missing data.

In the survey administration section, 79% did not describe financial incentives and 68% did not tell how they approached their participants. In the results section, 81% of the papers did not tell how respondents differed from the non-respondents.

### Quality of reporting in web-based surveys using Eysenbach et al.’s CHERRIES guidelines

We included 100 web-based survey studies using the methodology described in the flow chart in Fig 1. Using Eysenbach et al.’s updated CHERRIES guidelines, we assessed the reporting quality of those 100 studies and reported all the results in Table 2.

Most of the items here were under-reported. To highlight some of these items, in the survey administration section, more than 90% of the papers did not mention enough details regarding randomization of items of questionnaires, adaptive questioning, number of questionnaire items per page, number of screens (pages) in the questionnaire, completeness check, a non-response option or if a review step was used or not.

The same is evident with some aspects of response rates like the number of unique site visitors and the view rate. Also, most of the papers (98%) did not mention or did not prevent individuals from giving multiple entries either by using cookies or IP check, and most of them did not give satisfying details about their analysis, like how they handled incomplete questionnaires (77%) or if they used methods to adjust for the non-representative sample (94%).

## Discussion

Poor reporting of medical studies can critically jeopardize the integrity of medical knowledge synthesis.[16] It is recognized as a significant problem in clinical research that negatively impacts the progress of medical development.[10] We found that the majority of items in both sets of guidelines that we used in our assessment were under-reported. Our results confirm a potential gap in the reporting practices of survey-based studies, and highlight the necessity of constructing an updated comprehensive guideline that will help researchers and reviewers make better decisions when it comes to data collection by surveys.

Reporting guidelines are considered a vital tool to overcome the variance and incompleteness of presenting data in research studies.[17, 18] However, developing a guideline demands collaborative efforts and several investigations to produce a robust, high-quality and reliable guiding checklist.[19] We believe that this study is a prospective core for a future guideline that encompasses all poor-reporting issues in survey research.

In our review, many items of SURGE and CHERRIES guidelines are uncommonly reported. For web-based studies, these items include data protections, ethical considerations, methodology of survey administration, characteristics of the survey, completeness check, response rates, preventing multiple entries and statistical correction. For non-web-based studies they include validity and reliability of measurement tool, scoring procedures, sample size calculation, representativeness of the sample, mode of administration, incentives, who approached potential participants, completeness of the survey, non-response error, handling missing data, response rates and ethical considerations. Authors, who fail to adhere to these items, or any item listed in a certain guideline, might not be aware of the importance of these missed aspects, and the importance of following universal guidelines for reporting the results of their research.

In survey research, investigators can collect data using a preexisting validated tool, or a scale of their own development that serves their study outcomes. Generally, whatever tools of measurement researchers use, their scales are expected to be valid, reliable and consistent in measuring the concepts under investigation.[20, 21] In our study, only 21% of non-web-based studies discussed the validity and reliability, or both, of the tools used in their studies. This item is not listed in CHERRIES guidelines and as data was extracted according to their checklist, we did not evaluate it in our included web-based surveys. In addition, reporting the scoring procedures of scales, or providing the core questions of the survey can highly help other researchers build on current findings, and hence, increase the quality of current research. Reproduction and inter-studies comparison have been well-emphasized in medical research.[22, 23] Therefore, we believe that this is a noteworthy aspect of survey research, and that researchers should refer to it in their manuscripts for web based or non-web based surveys.

Survey research is not immune to bias. Certain types of misconduct can occur while recruiting a sample or distributing a survey. Researchers commonly seek a representative sample size to collect their data from. Inadequate sample recruitment can severely decrease the quality and accuracy of the study results.[24] Some researchers may fall into selection bias while attempting to reach an adequate representative sample, especially when they post online surveys on specifically targeted websites or when they send them to a limited mailing list, or when the research team fails to achieve proper randomization while recruiting research subjects. Ambiguity related to such kind of bias can be resolved with proper reporting of the subjects' recruitment process, sample frame and characteristics, sample size calculating techniques, and the representativeness of the sample. These data can help to assess the generalizability of the results in a certain population, which is crucial to determine the applicability of a study's results, and is recommended to be reported as well.[25]

Other types of bias can arise with distorted methodology. Careful consideration of the applied methods and the design of the questionnaire, with clear and detailed reporting of the study's strategy, can reduce the risk of misinterpretation and misuse of the research findings.[20] One of the important methodological calls that researchers make prior to initiating their data collection is the method of administration of the survey. These methods, whether online or offline, vary widely with many advantages and disadvantages for each one of them.[26] For instance, telephone interviews reduce the cost and speed up the collection process. However, they contribute to high risk of sample composition bias and affect the generalizability of results.[27, 28] Readers have the right to be informed of the way in which participants responded to the survey, and to assess the trustworthiness and risk of bias of the study. Data entry procedures for paper-based surveys, methods of survey advertisements for web-based surveys, and completeness check and preventing multiple-participations for both types, can also affect readers' judgment in regards to the study's proper conduct.

Some ethical concerns were raised with regard to survey research; whether it was paper-based or web-based.[29, 30] We have investigated the reporting of several aspects of research ethics in our review. Our results confirm potential under-reporting practices for ethical components in survey-based studies. Subjects' privacy has been questioned particularly in web-based research.[31] We believe that ensuring anonymity and reporting data protection measures can resolve these concerns. In addition, ethical controversies can be addressed with proper conduct and complete reporting of informed consent procedures, which are required prior to involving human subjects in any healthcare approach,[32] with incentives, which should be reasonable and avoid coercion of subjects into participation,[33] and non-response options, which are part of respecting subjects’ autonomy.[34] Overall, careful attention and complete reporting of ethical considerations can increase the transparency and credibility of any type of research,[10] including survey research.[35]

Low response rate can be an indicator of a possible non-respondent bias.[36] It can also decrease the generalizability of the results and affects the representativeness of the sample.[36, 37] Adaptive questioning, number of survey pages and items can contribute to low response rates.[38] Authors are urged to explicitly state the response rate and all related details that help editors and reviewers judge the quality of a research paper. In some cases, responses vary within the questionnaire items. Investigators are advised to define the cutoff where variance is acceptable and where it compels an omission of the whole survey. Defining incomplete questionnaires and statistical correction for missing items can help make better sense of the presented data and analyses. In this report, we emphasize the importance of complete, accurate, transparent and focused reporting of all items and details related to the characteristics, methodology, and outcomes of the survey-based studies.

Although our study systematically and thoroughly assessed the reporting quality of survey studies, whether online or offline, it poses some limitations. We only used PubMed for our search. Although it is one of the most popular and inclusive medical search engines, this might slightly affect the generalizability of our findings. Another limitation is that our sample did not include a sufficient number of non-English articles, or studies that used surveys as a secondary tool, like RCTs. Therefore, carrying out several comparisons of the difference of quality reporting between these variables was not possible. This issue can be addressed in future studies. In addition, although we have included two hundred studies in our analysis, we still think that studies with larger sample size can be more conclusive in terms of investigating the trends of reporting quality over the past years.

In summary, inconsistent survey designs and reporting practices can represent significant challenges in assessing the quality of evidence surveys resent. Omitting important information, such as the characteristics and development of the survey, methods of subjects' recruitment and survey administration, data management and presentation, and ethical considerations could conceal fallacies that would potentially invalidate the presented evidence. Population samples could be non-representative if they are subject to different types of bias, such as selection bias, interviewer bias, healthy-user bias, exclusion bias, and non-respondent bias. Researchers, editors and consumers must be able to critically assess all information related to an article and, accordingly, make informed decisions in various fields of science and health. We therefore recommend that the SURGE and CHERRIES checklists should be further studied, according to our findings, and reproduced as a universal guideline that serves as a standard quality-reporting instrument for researchers. Depending on our results and the resources that we have used to get this study done, and taking into concrete consideration the gaps that we detected, the challenges that we faced, and the pros and cons of each item, we have created a table that summarizes our findings, and combines all frequently-used items of CHERRIES and SURGE (appendix 1). Its primary aim is to provide all healthcare professionals and readers with a comprehensive summary that serves all types of survey-based studies and that bridges the gaps in available tools. In addition, since this set of items was not validated yet, it is thought to facilitate the creation and validation of new tools/checklists in the future. We finally urge medical journals to endorse well-defined guidelines and adopt high standards of reporting quality to increase research credibility and reduce scientific waste. We believe that working towards globally unifying research guidelines would not limit creativity, but will rather organize and structure evidence for a more efficient and practical consumption of research.

## Supporting information

S1 File Supplemental data sheet with 500 studies. List of all 500 texts that were selected. Studies included in the analysis as eligible studies are marked.
(XLSX)

S2 File Appendix. A comprehensive summary with all items that potentially assess the reporting quality of survey-based studies; the SUrvey Research Guideline and CHERRIES items were melted into one table that covers all aspects of web-based and non-web-based studies. This table is aimed to facilitate the creation and validation a combined checklist in the future.
(DOCX)

S3 File Final version of the data sheet. (XLSX)
