# Supplementary material for: Quality of reporting web-based and non-web-based survey studies: What authors, reviewers and consumers should consider
Source: PLoS One. 2018 Jun 18;13(6):e0194239. doi: 10.1371/journal.pone.0194239 (PMC6005542; doi:10.1371/journal.pone.0194239)
Supplement: S2 File — A comprehensive summary with all items that potentially assess the reporting quality of survey-based studies; the SUrvey Research Guideline and CHERRIES items were melted into one table that covers all aspects of web-based and non-web-based studies. This table is aimed to facilitate the creation and validation a combined checklist in the future. (DOCX) [file pone.0194239.s002.docx]

**Appendix 1.** A comprehensive summary with all items that potentially assess the reporting quality of survey-based studies; the SUrvey Research Guideline and CHERRIES items were melted into one table that covers all aspects of web-based and non-web-based studies. This table is aimed to facilitate the creation and validation a combined checklist in the future.

| Checklist Item | Explanation | Scoring procedures |
| --- | --- | --- |
| Title and abstract |  |  |
| Design of the study stated | Are the words 'questionnaire' or 'survey' stated in the title and/or abstract? | Both title and abstract |
|  |  | Either title or abstract |
|  |  | Not stated |
| Introduction |  |  |
| Background provided | Did the authors present a well-written background to their research? | Yes |
|  |  | No |
| Purpose/aim of paper explicitly stated | Did the authors identify a specific purpose, aim, goal, or objective of the study? | Yes |
|  |  | No |
| Tool of measurement |  |  |
| Description of the questionnaire | Did they provide access to the questionnaire items used in the study in either the article, appendices, or an online supplement? | Questionnaire provided |
|  |  | Core questions provided |
|  |  | One complete question provided |
|  |  | Questions not provided |
| Existing tool, psychometric properties presented | Existing tool = (the questionnaire was constructed by others and the authors just used it). Psychometric properties = (were the reliability and validity of the existing questionnaire mentioned?) | Yes |
|  |  | No |
|  |  | Not applicable |
| Existing tool, references to original work provided | Did they provide a reference to the existing tool (questionnaire) that they used? | Yes |
|  |  | No |
|  |  | Not applicable |
| New tool, procedures to develop and pre-test provided | Did studies that developed a novel questionnaire clearly describe the development process and/or describe the methods used to pre-test the tool? Have the usability and technical functionality of electronic questionnaires been tested before fielding them? | Yes |
|  |  | No |
|  |  | Not applicable |
| New tool, reliability and validity reported | Did they report the reliability and validity of the developed questionnaire? | Both |
|  |  | Reliability only |
|  |  | Validity only |
|  |  | Neither |
|  |  | Not applicable |
| Description of the scoring procedures provided | Did papers which used survey instruments that required scoring provide a description of the scoring procedures? | Yes |
|  |  | No |
|  |  | Not applicable |
| Recruitment process and sample description |  |  |
| Description of survey population and sample frame | Survey population = the main target of the study. Sample frame = the methods they used to reach this target. For instance, if a study wanted to detect the prevalence of hypertension in elderly people above 65 years old, and researchers went to 10 elderly care centers and randomly recruited 50 old men from the registry of each of those centers. This means that our survey population is elderly people above 65, and the sample frame is the 10 care centers registry. | Both |
|  |  | Survey population |
|  |  | Sample frame |
|  |  | Neither |
| Description of representativeness of the sample | Is a description of whether the sample will represent the whole population provided? | Yes |
|  |  | No |
| Sample size calculation or rationale/justification presented | Did they mention a description of their sample size calculation, such as providing a formula or a rationale? | Yes |
|  |  | No |
| Open survey versus closed survey* | An “open survey” is a survey that is available for each visitor of a site, while a closed survey is only open to a sample which the investigator knows (password-protected surveys and surveys in which participants are invited by individual survey links). | Open Survey |
|  |  | Closed Survey |
|  |  | Not mentioned |
| Advertising the survey | How/where was the survey announced or advertised? It is important to know the wording of the announcement as it will heavily influence who chooses to participate. Ideally, the survey announcement should be published as an appendix. | social media |
|  |  | Mixed |
|  |  | offline media (newspapers, letter of invitations)= |
|  |  | online mailing lists |
|  |  | banner ads |
|  |  | not mentioned |
| Incentives | Were any incentives offered (e.g., monetary, prizes, or non-monetary incentives such as an offer to provide the survey results)? | Mentioned with reporting the value of the incentive |
|  |  | Mentioned without reporting the value of the incentive |
|  |  | Not mentioned |
| **Survey Administration** |  |  |
| Mode of administration | Did the authors specify how they administered the survey to the participant? | In person |
|  |  | Website link |
|  |  | E-mail |
|  |  | Telephone |
|  |  | Mail |
|  |  | Mixed |
|  |  | Not mentioned |
| Information on the type and number of contacts provided | This refers to the initial contact between the participants and the researchers; type (i.e. phone, e-mail, postal mail, online/offline advertisement), number of contacts (i.e. how many times they tried to contact them?) | Type and number |
|  |  | Type only |
|  |  | No information |
| Description of who approached potential participants | Description of who was identified as the organization/group urging potential research subjects for their participation in the survey. Did they mention the group giving money for participants to enter the study or the group who convinced them to participate and explained the study to them? | Yes |
|  |  | No |
|  |  | Not applicable |
| Context* | Description of the Web site (for mailing list/newsgroup) on which the survey was posted. What is the Web site about, who is visiting it, what are visitors normally looking for? Discuss to what degree the content of the Web site could pre-select the sample or influence the results. For example, a survey about vaccination on an anti-immunization Website will have different results from a Web survey conducted on a government Website | Given in details |
|  |  | Some partial information |
|  |  | No description |
|  |  | Not applicable |
| Preventing multiple entries from the same individual* | This is mostly concerned with open surveys. Did they indicate whether cookies were used to assign a unique user identifier to each client's computer? If so, did they mention the page on which the cookie was set and read, and for how long was the cookie valid? Were duplicate entries avoided by preventing users' access to the survey twice? or were duplicate database entries having the same user ID eliminated before analysis? In the latter case, which entries were kept for analysis (eg, the first entry or the most recent)?  Did they indicate whether the IP address of the client's computer was used to identify potential duplicate entries from the same user? If so, did they mention the period of time for which no two entries from the same IP address were allowed (e.g., 24 hours)? Were duplicate entries avoided by preventing users with the same IP address access to the survey twice; or were duplicate database entries having the same IP address within a given period of time eliminated before analysis? If the latter, which entries were kept for analysis (eg, the first entry or the most recent)?  Did they indicate whether other techniques to analyze the log file for identification of multiple entries were used?  In “closed” (non-open) surveys, users need to login first and it is easier to prevent duplicate entries from the same user. Did they describe how this was done? For example, was the survey never displayed a second time once the user had filled it in, or was the username stored together with the survey results and later eliminated? If the latter, which entries were kept for analysis (eg, the first entry or the most recent)? | Cookies |
|  |  | IP check |
|  |  | Log file analysis |
|  |  | Registration |
|  |  | Other techniques |
|  |  | Not mentioned |
| Mandatory/voluntary | Was it a mandatory survey to be filled in by every visitor who wanted to enter the Website, or was it a voluntary survey? | Mandatory |
|  |  | Voluntary |
|  |  | Not mentioned |
| Time/Date | In what timeframe were the data collected? | Mentioned |
|  |  | Not mentioned |
| Randomization of items or questionnaires | To prevent biases items can be randomized or alternated. | Randomized or alternated |
|  |  | Not randomized or not mentioned |
| Completeness check | It is technically possible to do consistency or completeness checks before the questionnaire is submitted. Was this done? If yes, how (usually JAVAScript)? An alternative is to check for completeness after the questionnaire has been submitted (and highlight mandatory items). If this has been done, it should be reported. All items should provide a non-response option such as “not applicable” or “rather not say”, and selection of one response option should be enforced. | Consistency or completeness checks were used before the questionnaire is submitted |
|  |  | After it was submitted |
|  |  | Not mentioned |
|  |  | Not applicable |
| Review step* | Did they state whether respondents were able to review and change their answers? (e.g., through a "Back" button or a "Review step" which displays a summary of the responses and asks the respondents if they are correct). | Mentioned |
|  |  | Not used or not mentioned |
| Response rates |  |  |
| Response rate reported | For web-based surveys, authors should count the unique number of visitors who visit the first page of the survey (or the informed consents page, if present) divided by the number of people who filled in the first survey page (or agreed to participate). This can also be called “recruitment” rate. | Yes, defined |
|  |  | Yes, not defined |
|  |  | Partial information |
|  |  | No information |
| Completion rate (Ratio agreed to participate/finished survey) | The number of people agreeing to participate (or submitting the first survey page) divided by the number of people submitting the last questionnaire page. This is only relevant if there is a separate “informed consent” page or if the survey goes over several pages. This is a measure for attrition. Note that “completion” can involve leaving questionnaire items blank. This is not a measure for how completely questionnaires were filled in. (If you need a measure for this, use the word “completeness rate”.) | Yes, defined |
|  |  | Yes, not defined |
|  |  | Partial information |
|  |  | No information |
| Unique site visitor* | If view rates or participation rates are provided, authors need to define how you determined a unique visitor. There are different techniques available, based on IP addresses or cookies or both. | Mentioned |
|  |  | Not mentioned |
| Adaptive questioning | Did they use adaptive questioning (certain items are only conditionally displayed based on responses to other items) to reduce number and complexity of the questions. Can affect the response rate. | Used adoptive questioning |
|  |  | Did not use it or did not mention it |
| Number of Items | What was the number of questionnaire items per page? The number of items is an important factor for the completion rate. | Mentioned |
|  |  | Not mentioned |
| Analysis |  |  |
| Methods of data analysis | Was a description of the variables that were analyzed, how they were manipulated, and the statistical methods that were used provided? | Adequate |
|  |  | Inadequate |
|  |  | No description |
| Method for analysis of nonresponse error provided | _ | Yes |
|  |  | No |
| Definitions for complete versus partial completions provided | Was a definition or cut-off limit for partial completion of questionnaires provided? | Yes |
|  |  | No |
| Methods for handling item missing data provided | Were the methods for handling item missing data provided? | Yes |
|  |  | No |
| Questionnaires submitted with an atypical timestamp | Some investigators may measure the time people needed to fill in a questionnaire and exclude questionnaires that were submitted too soon. Did they specify the timeframe that was used as a cut-off point, and describe how this point was determined? | The timeframe that was used as a cut-off point was given and described why |
|  |  | Given but did not mention the reason |
|  |  | Not mentioned |
| Statistical correction | Did they indicate whether any methods such as weighting of items or propensity scores have been used to adjust for the non-representative sample? | Methods to adjust for the non-representative sample was given and described |
|  |  | Mentioned but not well-described |
|  |  | Not mentioned |
| Results |  |  |
| All respondents accounted for | Did they report the sample disposition (i.e., describing the number of complete and partial returned questionnaires according to the number of potential participants known to be eligible, of unknown eligibility, or known to be ineligible)? | Yes |
|  |  | No |
| Information on how non-respondents differ from respondents provided | _ | Yes |
|  |  | Issue addressed |
|  |  | No information |
| Results clearly presented | _ | Yes – complete |
|  |  | Yes – partial |
|  |  | No |
| Results address objectives | _ | Yes |
|  |  | No |
| Discussion |  |  |
| Results summarized referencing study objectives | _ | Yes |
|  |  | No |
| Strengths of the study stated | _ | Yes |
|  |  | No |
| Limitations of the study stated | _ | Yes |
|  |  | No |
| Generalizability of results discussed | Did they include any discussion on the generalizability of their results? | Yes |
|  |  | No |
| **Ethical Quality Indicators** |  |  |
| Study funding reported |  | Yes |
|  |  | No |
| Research Ethics Board (REB) review reported |  | Yes |
|  |  | Reported REB exempt |
|  |  | No |
| Subject consent procedures reported |  | Yes |
|  |  | Reported waiver of informed consent |
|  |  | No |
| Data protection* | If any personal information was collected or stored, did they describe what mechanisms were used to protect unauthorized access? | If authors collected or stored any personal information, they gave the mechanisms used to protect unauthorized access. |
|  |  | Mechanisms used are not given |
| The (*) symbol refer to items that are more concerned with web-based studies. | | |
